# The burden of submicroscopic and asymptomatic malaria in India revealed from epidemiology studies at three varied transmission sites in India

**DOI:** 10.1038/s41598-019-53386-w

**Published:** 2019-11-19

**Authors:** Anna Maria van Eijk, Patrick L. Sutton, Lalitha Ramanathapuram, Steven A. Sullivan, Deena Kanagaraj, G. Sri Lakshmi Priya, Sangamithra Ravishankaran, Aswin Asokan, V. Sangeetha, Pavitra N. Rao, Samuel C. Wassmer, Nikunj Tandel, Ankita Patel, Nisha Desai, Sandhya Choubey, Syed Zeeshan Ali, Punam Barla, Rajashri Rani Oraon, Stuti Mohanty, Shobhna Mishra, Sonal Kale, Nabamita Bandyopadhyay, Prashant K. Mallick, Jonathan Huck, Neena Valecha, Om P. Singh, K. Pradhan, Ranvir Singh, S. K. Sharma, Harish C. Srivastava, Jane M. Carlton, Alex Eapen

**Affiliations:** 1 Center for Genomics and Systems Biology, Department of Biology, New York University, New York, NY 10003 USA; 2 Indian Council of Medical Research - National Institute of Malaria Research, IDVC Field Unit, National Institute of Epidemiology Campus, Ayapakkam, Chennai, Tamil Nadu India; 3 Department of Zoology, Madras Christian College, University of Madras, Tambaram, Chennai 600 059 India; 4 Indian Council of Medical Research - National Institute of Malaria Research Field Unit, Civil Hospital, Nadiad, Gujarat India; 5 Jigyansha, International Center of Excellence for Malaria Research, Sector 1, Rourkela, Odisha India; 6 Indian Council of Medical Research, National Institute of Malaria Research, Dwarka Sector 8, New Delhi, India; 7 Department of Geography Arthur Lewis Building, The University of Manchester, Manchester, England; 8 Present Address: GlaxoSmithKline, 5 Moore Drive, PO Box 13398, RTP, Raleigh, NC 27709-3398 United States; 9 Present Address: Memorial Sloan Kettering Cancer Center, 1275 York Avenue, New York, NY 10065 USA; 10 Present Address: London School of Hygiene and Tropical Medicine, Keppel St, London, WC1E 7HT United Kingdom; 11 Present Address: Institute of Science, Nirma University, Gujarat, 382481 India

**Keywords:** Malaria, Epidemiology

## Abstract

Malaria in India, while decreasing, remains a serious public health problem, and the contribution of submicroscopic and asymptomatic infections to its persistence is poorly understood. We conducted community surveys and clinic studies at three sites in India differing in their eco-epidemiologies: Chennai (Tamil Nadu), Nadiad (Gujarat), and Rourkela (Odisha), during 2012–2015. A total of 6,645 subject blood samples were collected for *Plasmodium* diagnosis by microscopy and PCR, and an extensive clinical questionnaire completed. Malaria prevalence ranged from 3–8% by PCR in community surveys (24 infections in Chennai, 56 in Nadiad, 101 in Rourkela), with *Plasmodium vivax* dominating in Chennai (70.8%) and Nadiad (67.9%), and *Plasmodium falciparum* in Rourkela (77.3%). A proportional high burden of asymptomatic and submicroscopic infections was detected in community surveys in Chennai (71% and 71%, respectively, 17 infections for both) and Rourkela (64% and 31%, 65 and 31 infections, respectively). In clinic studies, a proportional high burden of infections was identified as submicroscopic in Rourkela (45%, 42 infections) and Chennai (19%, 42 infections). In the community surveys, anemia and fever were significantly more common among microscopic than submicroscopic infections. Exploratory spatial analysis identified a number of potential malaria hotspots at all three sites. There is a considerable burden of submicroscopic and asymptomatic malaria in malarious regions in India, which may act as a reservoir with implications for malaria elimination strategies.

## Introduction

Malaria remains a major public health burden in the south-east Asia region, and approximately 80% of malaria cases of the nine countries in this region occur in India^1^. As the seventh largest and second most populous country in the world, the subcontinent contributes nearly half of the global population at risk of *Plasmodium vivax*^1^. Malaria surveillance is primarily conducted by active case detection by accredited social health activists (ASHAs) and passive case detection at Primary Health Centres (PHCs), Community Health Centres (CHCs), or malaria clinics. These surveillance programs included over 120 million blood smear examinations in 2014, equivalent to sampling ~10% of the population^2^. Although blood smear microscopy remains the most common diagnostic method, the number of malaria rapid diagnostic tests (RDTs) supplied by local and regional manufacturers has more than doubled since 2010^2^. Molecular methods such as polymerase chain reaction (PCR) are expensive and require sophisticated laboratory equipment, making them unfeasible for routine diagnosis.

India has joined other countries on the path to malaria elimination and has recently developed a five-year *National Strategic Plan for Malaria Elimination in India 2017–2022*, whose goal is to eliminate malaria in states with low level malaria (<1 annual parasite incidence) or bring states with higher levels of malaria to pre-elimination levels by 2022^3^. The epidemiology of malaria in India is complex. Both *P*. *falciparum* and *P*. *vivax* can be present, with falciparum malaria tending to dominate in the eastern and northeastern provinces, and *P*. *vivax* in other parts of the country. Malaria elimination strategies in India will need to be fine-tuned according to the endemicity at the state and district levels. For this purpose, it is important to know the real burden of malaria, *i*.*e*., not only what is routinely detected, but also what is asymptomatic (infections in people who do not feel sick and so who do not visit a clinic for diagnosis or treatment), or below the level of detection of the current common diagnostic tools of microscopy (submicroscopic) or RDTs. Recent reviews have shown the importance of submicroscopic infections for both *P*. *falciparum* and *P*. *vivax*^4,5^, although studies from India are lacking. To understand the local epidemiology of malaria in India and the submicroscopic and asymptomatic burden further, we conducted a series of cross-sectional and clinic-based epidemiology studies at three sites in India with different malaria endemicities as part of an International Center for Excellence for Malaria Research^6^. A better understanding of the epidemiology of malaria in India is critical for allocating resources and assessing the impact of malaria control efforts. Here we describe the prevalence of malaria, species of *Plasmodium* parasite, presence of submicroscopic and asymptomatic infections, and identify hotspots, as well as assess the factors associated with malaria for each setting.

## Results

### Characteristics of the study populations

**Figure 1:**
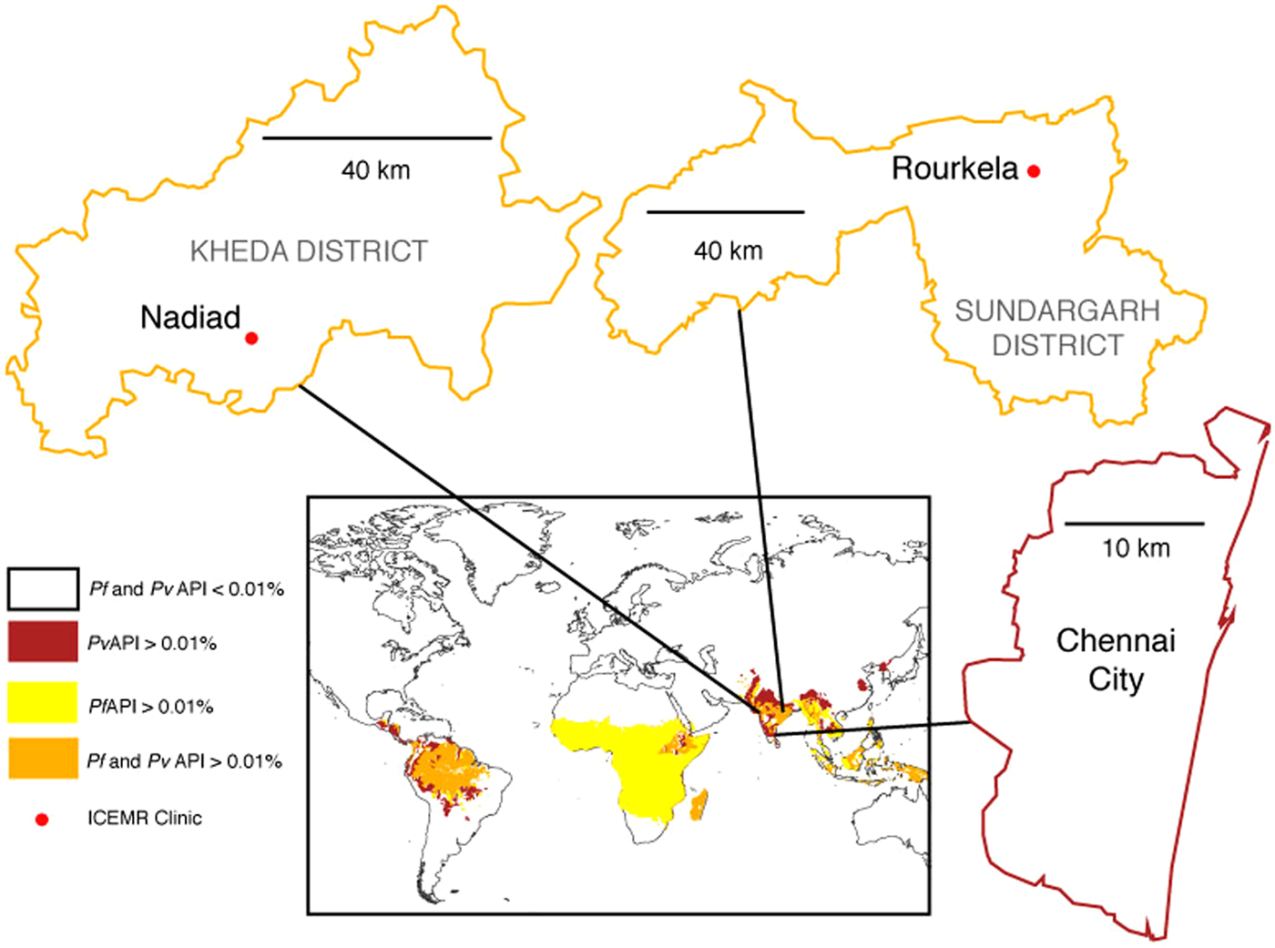
Location of three study sites in India. The inset shows a global map of malaria endemicity as annual parasite incidence (API), adapted from the Malaria MAP Project. The three study sites are indicated in the west, east and south of India.

Our three study sites were chosen because of their different eco-epidemiologies: Chennai, in the southern state of Tamil Nadu; Nadiad in the western state of Gujarat,; and Rourkela in the eastern state of Odisha (Fig. 1). We conducted two different types of epidemiology study: cross-sectional surveys in the communities, which enrolled 928 subjects in Chennai, 796 subjects in Nadiad, and 1,307 subjects in Rourkela (Table 1), and studies at local malaria clinics, which enrolled 1,054 subjects in Chennai, 685 subjects in Nadiad, and 1,875 subjects in Rourkela. After enrolment of all 6,645 participants, a detailed clinical questionnaire was completed, and a blood sample collected for *Plasmodium* diagnosis by microscopy, rapid diagnostic test (RDT), and PCR, and for testing of hemoglobin level. The study populations enrolled at the three sites differed in their characteristics (Table 1), *e*.*g*., males were more likely to take part in clinic studies compared to the community surveys; in the Rourkela site there were more participants <15 years, and of lowest educational level compared to the other sites. Anemia was common among participants (35.4%, 62.5%, and 48.8% of participants in community surveys in Chennai, Nadiad and Rourkela, respectively).

**Table 1:** Characteristics of participants by location and type of study at three study sites in India, 2012–2015.

	Community surveys (weighted by age and gender)			Clinic studies		
	Chennai % or mean (95% CI)	Nadiad % or mean (95% CI)	Rourkela % or mean (95% CI)	Chennai % or mean (95% CI)	Nadiad % or mean (95% CI)	Rourkela % or mean (95% CI)
Number of participants	928	796	1307	1054	685	1875
Time period	Dec 12-Oct 14	May 13-Sept 14	Jan 13-Sep 14	Apr 12-Mar 15	Jan 13-Apr 15	Apr 12-Apr 15
Median age, interquartile range	29.0, 17.0–43.0	30.0, 17.0–45.0	25.0, 12.0–40.0	30.0, 21.0–42.0	25.0, 13.0–40.0	28.0, 12.0–43.0
Age groups (%)						
<5 years	6.3 (4.3–9.2)	5.4 (3.5–8.3)	8.8 (7.5–10.3)*	0.9 (0.5–1.8)	3.8 (2.6–5.5)	8.6 (7.4–10.0)^†^
5–14 years	14.0 (11.7–16.7)	15.4 (13.0–18.1)	21.3 (19.3–23.5)	7.7 (6.2–9.5)	23.1 (20.1–26.4)	20.8 (19.0–22.7)
15+ years	79.7 (76.3–82.7)	79.2 (75.8–82.2)	69.9 (67.5–72.2)	91.4 (89.5–92.9)	73.1 (69.7–76.3)	70.6 (68.5–72.6)
Male	50.4 (46.7–54.0)	51.6 (48.0–55.2)	50.5 (47.8–53.2)	68.0 (65.1–70.8)^‡^	60.4 (56.7–64.0)	57.4 (55.1–59.6)
**History and symptoms**						
Documented fever	3.4 (2.4–4.9)^‡^	6.1 (4.7–8.0)	7.7 (6.5–9.2)	34.1 (31.3–37.0)**	29.1 (25.8–32.6)	32.6 (30.5–34.8)
History of fever last 2 days	10.9 (8.7–13.7)	22.4 (19.6–25.5)	7.4 (6.2–8.8)^†^	95.0 (93.5–96.1)	67.4 (63.8–70.9)	93.0 (91.7–94.0)^†^
History of fever or documented fever	11.3 (9.0–14.1)	24.5 (21.6–27.7)^§^	12.4 (10.8–14.1)	96.0 (94.6–97.0)	77.8 (74.5–80.8)	94.1 (93.0–95.1)^†^
History of malaria last year	5.9 (4.5–7.6)	4.6 (3.1–6.7)	25.9 (23.6–28.2)*	16.6 (14.5–19.0)^‡^	7.3 (5.6–9.5)	6.7 (5.6–7.9)
History of travel past 2 wks	12.5 (10.3–15.1)	11.1 (9.0–13.7)	2.6 (1.9–3.6)*	16.4 (14.3–18.8)	13.6 (11.2–16.4)	23.7 (21.8–25.6)*
Antimalarial use past 2 wks	0.3 (0.1–0.8)	5.7 (4.2–7.7)	1.6 (1.1–2.4)^†^	2.3 (1.6–3.4)	0.6 (0.2–1.6)^§^	2.5 (1.8–3.3)
Use of ITNs	0	0.6 (0.3–1.5)	28.4 (26.2–30.9)^†^	0	0	0.1 (0.0–0.4)
Use of repellents^††^	45.6 (42.0–49.4)	39.5 (36.1–43.1)	32.5 (29.8–35.3)^†^	33.7 (30.9–36.6)	50.2 (46.5–54.0)	77.9 (76.0–79.7)^†^
Anemia (%)^‡‡^	35.4 (32.2–38.8)	62.5 (59.0–65.9)	48.8 (46.0–51.7)^†^	26.5 (23.9–29.2)	54.4 (50.6–58.1)	36.1 (33.9–38.3)^†^

CI: confidence interval, ITN: insecticide treated net (long lasting insecticide treated net or a net treated within the last 6 months), wks: weeks.

^*^P < 0.05 comparing Rourkela to Chennai and Nadiad.

^†^P < 0.05 comparing to each other.

^‡^P < 0.05 comparing Chennai to Nadiad and Rourkela.

^§^P < 0.05 comparing Nadiad to Chennai and Rourkela.

^**^P < 0.05 comparing Chennai to Nadiad.

^††^Repellents include the use of coils, vaporizers, mats or creams for the prevention of mosquito annoyance.

^‡‡^Age and gender appropriate definition: Hemoglobin < 11 g/dl if age < 5 years, <11.5 g/dl if age ≥ 5 and <12 years, <12 g/dl if age > 12 and age < 15, <12 g/dl if age ≥ 15 and female, and <13 g/dl if ≥15 and male.

### Malaria prevalence and symptomatic infections in the community surveys

**Figure 2:**
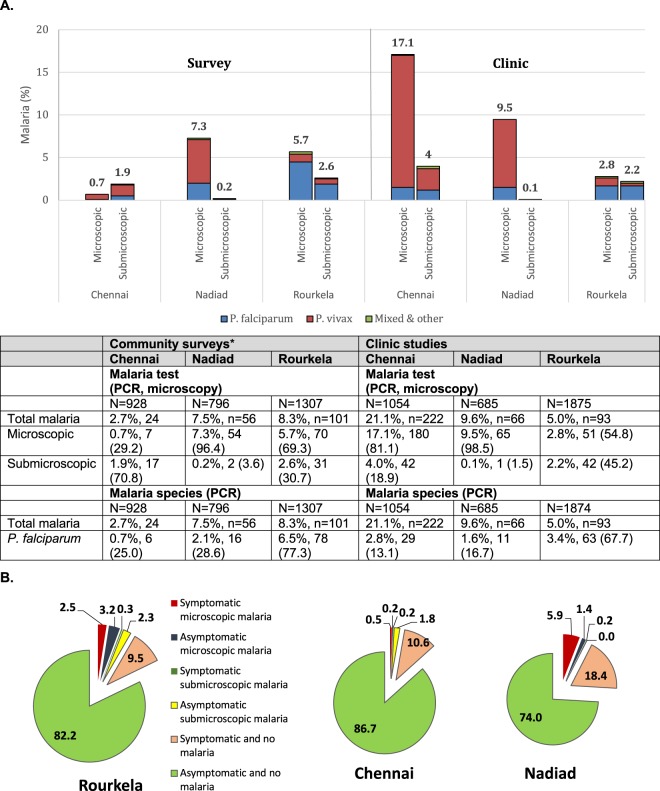
Malaria at three sites in India, 2012–2015. (**A**) Malaria in community surveys and clinic studies at the three sites. *The first percentages in the columns are the percentages of malaria (weighted by age and gender for the community surveys); the numbers after the percentages indicate the absolute number of infections in the group, and those in brackets represent the percentage in this group of all infections. Symptomatic malaria was defined as documented fever (a body temperature ≥37.5 °C) or a history of fever in the past 48 hours. (**B**) Symptomatic and asymptomatic malaria infections as percentages (weighted for age and sex) in community surveys at the three sites. For actual numbers and *Plasmodium* species, see Supplemental Table S1.

We found the malaria prevalence (measured by PCR, weighted by gender and age) in our community surveys to be 2.7% in Chennai, 7.5% in Nadiad, and 8.3% in Rourkela (Fig. 2), with *P*. *falciparum* the dominant species in Rourkela and *P*. *vivax* more common at the other sites. Risk factors identified for malaria (using generalized linear regression with a log link and binomial distribution) included (1) rainy season (as measured by PCR and microscopy) at all sites (Supplemental Table S2 and Fig. S2), and (2) male gender, associated with *P*. *falciparum* (detected by PCR) in Chennai and Rourkela (Supplemental Table S2). In Rourkela, where 30.1% of enrolled participants were <15 years old, *P*. *vivax* diagnosed by PCR was more common among children <5 years old, whereas *P*. *falciparum* was more common in the age group 5–14 years of age compared to persons ≥15 years (Supplemental Table S2).

### High burden of asymptomatic and submicroscopic infections in the community survey

We found that asymptomatic infections detected by PCR made up 70.8% of all malaria infections in Chennai (n = 17 asymptomatic infections, 2 (11.8%) microscopic and 15 or 88.2% submicroscopic), 64.4% of all malaria infections in Rourkela (n = 65 asymptomatic infections, 38 or 58.5% microscopic and 27 or 41.5% submicroscopic), and 21.4% of all malaria infections in Nadiad (n = 12 asymptomatic infections, all microscopic; Fig. 2). Symptomatic *P*. *falciparum* infections had significantly higher parasite densities compared to asymptomatic infections in Rourkela; no such differences were detected for the other sites (although numbers were low; Supplemental Table S3). Asymptomatic *P*. *falciparum* infections were more common among males in Chennai, whereas recent antimalarial use and travel were associated with asymptomatic *P*. *vivax* in Nadiad (Supplemental Table S2). In Rourkela, asymptomatic infections were significantly more common for *P*. *vivax* compared to *P*. *falciparum* (85.4% vs. 62.0%, *P* = 0.034). Additionally, asymptomatic *P*. *falciparum* infections were associated with the rainy season, being male, and ITN (insecticide treated net) use, and asymptomatic *P*. *vivax* infections with repellent (protective) and recent antimalarial use (Supplemental Table S2).

Similarly, submicroscopic infections were detected in 70.8% of the cases in Chennai (17 infections), and 30.7% of the cases in Rourkela (31 infections), although they were not species-specific (Fig. 2; in Nadiad, all but two *Plasmodium* infections were detected by microscopy). Submicroscopic *P*. *falciparum* infections were more common among males at both sites, and submicroscopic *P*. *vivax* infections were associated with the rainy season and age <15 years compared to age ≥15 years in Rourkela (Supplemental Table S2).

**Figure 3:**
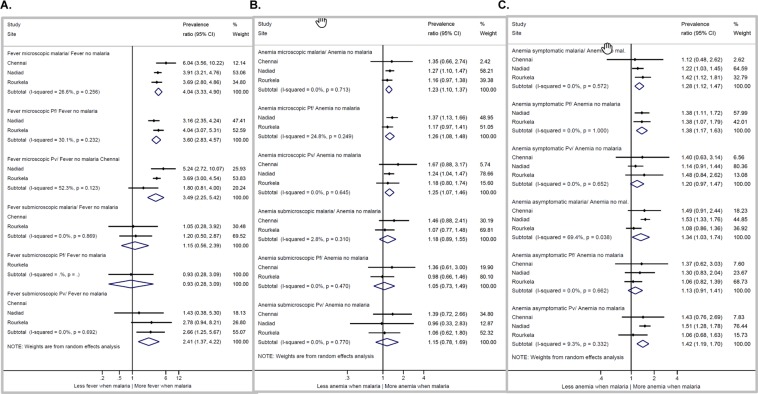
Associations between malaria and fever or anemia at three sites in India, 2012–2015. When a study site was not included, the site did not have enough information for this analysis. (**A**) The association between fever and microscopic/submicroscopic malaria from community surveys. Chennai not included: only one person with microscopic symptomatic *P*. *falciparum* infection, and six persons with submicroscopic asymptomatic *P*. *falciparum* infections (0 symptomatic submicroscopic persons). Nadiad not included: two persons with submicroscopic symptomatic malaria infections (1 *P*. *falciparum* and 1 *P*. *vivax*), 0 with asymptomatic malaria. Fever defined as a history of fever in the past 48 hours or documented fever (≥37.5 °C), malaria parasites detected by PCR. (**B**) The association between anemia and microscopic/submicroscopic malaria from community surveys. Chennai not included: only 1 person with microscopic malaria (*P*. *falciparum*) without anemia; Nadiad not included; only 1 person with submicroscopic malaria (*P*. *falciparum*) with anemia. (**C**) The association between anemia and symptomatic and asymptomatic malaria from community surveys. Chennai not included: only one person with symptomatic *P*. *falciparum* infection without anemia.

Effects of type of malaria infections in the community studies are reported in Fig. 3. First, malaria infections overall and *P*. *falciparum* infections detected by microscopy were typically associated with symptoms of fever, compared to persons without malaria as determined by PCR or microscopy, in contrast to submicroscopic infections (Fig. 3A); a similar pattern was seen for anemia (Fig. 3B). Second, the effect of symptomatic and asymptomatic malaria on anemia differed by site, with asymptomatic infections and symptomatic malaria associated with increased risk of anemia compared to no malaria in Nadiad, and only symptomatic malaria a risk factor for anemia in Rourkela (Fig. 3C). Finally, there was no significant difference in proportion of gametocytaemia comparing symptomatic versus asymptomatic *P*. *falciparum* infections, although in Rourkela they were more common among asymptomatic infections (5/54 vs. 2/37 in symptomatic infections, Supplemental Table S3). The majority of *P*. *vivax* infections contained gametocytes as determined by microscopy, except for in Rourkela (Supplemental Table S3).

### Mixed *Plasmodium* species infections were rare

We detected very few mixed *P*. *vivax and P*. *falciparum* infections across the community studies by PCR: one in Chennai, two in Nadiad and 5 in Rourkela (Supplemental Table S1). Mixed infections were more common in participants <15 years (6/627 [1%] vs. 2/2407 among ≥15 years [0.08%], Fisher’s exact test P = 0.001) in the community studies, but they were not more likely to be symptomatic compared to infections with one species (2/8 [25.0%] vs. 85/173 [49.1%], P = 0.182). Across the community surveys, participants with *P*. *falciparum* infections were almost four times more likely to be diagnosed with an additional *P*. *vivax* infection compared to persons without a *P*. *falciparum* infection (pooled prevalence ratio unweighted data 3.80, 95% CI 1.84–7.83, I^2^ 0%; Supplemental Fig. S4).

### Exploratory Spatial Data Analysis reveals malaria hot-spots in community studies

**Figure 4:**
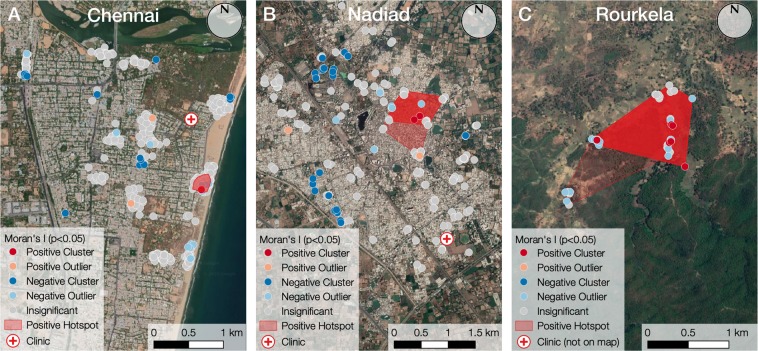
Examples of positive clusters of *Plasmodium* infections in community studies identified by PCR at sites in (**A**) Chennai, (**B**) Nadiad and (**C**) Rourkela. The LISA classification (positive or negative cluster or outlier) of each point is shown, and refers to the neighbourhood around each point, and not simply to the individual point. Red neighbourhood polygons (convex hulls constructed around all of the neighbours of a given point) are shown for each significant positive cluster and mark the maximum enclosing boundary of the ‘positive hotspot’ of *Plasmodium* infection, representing an area of interest (AOI) for further investigation. Satellite Imagery via Google Maps. Imagery © 2018 VNES/Airbus, Digital Globe, Landsat/Copernicus.

Using Exploratory Spatial Data Analysis (ESDA)^7^, we determined the presence, strength, and direction of spatial autocorrelation globally (across the entire dataset) and locally (within the immediate surroundings of each data point), of *Plasmodium* infections identified by PCR in the community studies. Global and Local Moran’s *I* analyses identified areas where greater concentrations of malaria occurred than would be expected from a random distribution of cases (Fig. 4). The Chennai (Fig. 4A), Nadiad (Fig. 4B) and Rourkela (Fig. 4C) sites exhibited *I* values of −0.008 (*p* = 0.2253), 0.125 (*p* = 0.0014), and 0.049 (*p* = 0.0002) respectively (pseudo-significance *p* values simulated using 9999 random permutations), indicating that Nadiad and Rourkela exhibit overall clustering (significant at the 5% level), whereas Chennai exhibited overall dispersal (not significant at the 5% level). This is notable because a vector-borne disease such as malaria typically would be expected to exhibit a strong clustered autocorrelation due to the spatial relationships between cases and the transmission of the parasite by *Anopheles* mosquito^8^.

Since local patterns may exist even if not evident across a dataset^7^, a single illustrative site was characterized further from each of the locations. In Chennai (Fig. 4A), two positive clusters (significant to the 5% level) were identified from 887 georeferenced PCR results in an area of 0.046 km^2^. The hotspots for these clusters overlapped entirely and provide a single area of interest (AOI) for further investigation, comprising an area known for housing fishermen in close proximity to the Besant Nagar beach. In Nadiad (Fig. 4B), four positive clusters (significant to the 5% level) were identified from 409 georeferenced PCR results. The hotspot polygons derived from the respective neighbourhoods of these clusters also overlapped, with three aligning perfectly in an area of 0.383 km^2^, and the fourth overlapping partially with an area of 0.503 km^2^. This delineated a single large AOI containing tightly packed high-rise housing interspersed with commercial buildings. Finally, in Rourkela (Fig. 4C), twelve positive clusters (significant to the 5% level) ranging in area from 0.456 km^2^ to 0.761 km^2^ were identified from 652 georeferenced PCR results. Again, the hotspot polygons overlapped to form a single AOI, encompassing a small number of rural dwellings in close proximity to forest, located either along roads or in small clusters, and two large areas of scrubland and several fields that exist between groups of settlements.

### Malaria and risk in clinic studies

In our clinic studies (passive case detection), malaria prevalence as detected by PCR was 21.1% (222 infections) in Chennai, 9.6% (66 infections) in Nadiad, and 5.0% (93 infections) in Rourkela; submicroscopic infections were 18.9% (42 infections) in Chennai, 1.5% (1 infection) in Nadiad, and 45.2% (42 infections) in Rourkela of the total malaria detected (Fig. 2). Common risk factors for malaria included being male, the rainy season, and a history of malaria in the past year (Supplemental Table S4). Although malaria by PCR overall was associated with anemia (pooled estimate 1.41, 95% CI 1.11–1.80 for any species) by site and species, this was only significant for microscopic *P*. *falciparum* in Nadiad, and for *P*. *vivax* in Chennai, and not for submicroscopic malaria (Supplemental Fig. S3).

## Discussion

To gain an understanding of the local epidemiology and burden of malaria in India, we conducted active and passive case detection studies in community and clinic settings at three sites. In the community (*i*.*e*., active) surveys, the point prevalence was low in all sites, with *P*. *vivax* dominating in Chennai and Nadiad, and *P*. *falciparum* in Rourkela, confirming the species distribution historically reported. In Chennai the majority of infections were submicroscopic (17 or 70.8%) and asymptomatic (17 or 70.8%), and similarly in Rourkela most infections were asymptomatic (65 or 64.4%) and 31 (35.6%) were not detected by microscopy. In contrast, Nadiad subjects that were asymptomatic (12 or 21.4%) or submicroscopic (2 or 3.6%) constituted only a minority of infections detected by community surveys. In our clinic studies, the majority of malaria infections were detectable by microscopy. However, approximately one in five infections in Chennai and two in five infections in Rourkela were submicroscopic, with troubling implications for malaria treatment and reservoirs of transmission. Moreover, just like symptomatic infections, asymptomatic infections detected in the community survey in Nadiad were associated with a significantly increased risk of anemia compared to persons without *Plasmodium* infections.

Very few surveys in India have used molecular methods such as PCR to assess the submicroscopic burden of malaria, and in those cases (as in our study) the results were variable and dependent upon the setting. For example, a survey in June 2012 in Purulia district, West Bengal, identified 6.9% *P*. *falciparum* infections by microscopy, 8.1% P. *falciparum* infections by PCR, and 1.2% submicroscopic infections^9^ among 963 participants; whereas a survey in the low transmission season of 2014 among 133 persons using insecticide treated nets in Missamari, Assam, detected no infections by PCR, microscopy, or rapid diagnostic test^10^. A study in 2007–2008 among pregnant women in Chhattisgarh, a state neighbouring Odisha, noted that the majority of infections detected in the antenatal clinic and in the placenta at delivery were submicroscopic (antenatal clinic 3.4% by PCR, 70.6% submicroscopic; placenta 4.2% by PCR, 73.8% submicroscopic), but no association with anemia was detected^11^. In three villages in Chhattisgarh, a survey in 2016 during the low transmission season detected 10% subpatent and 18% asymptomatic malaria^12^. A high submicroscopic or asymptomatic burden has been reported in studies elsewhere in Asia^4,13,14^; and a recent study in three southeast Asian countries found four times higher prevalence of submicroscopic *Plasmodium* infections with a sensitive PCR method compared with microscopy, indicating that the burden may still be underestimated^15^. We could retrieve few studies reporting on the effects of submicroscopic malaria from areas with both malaria parasite species: in Brazil, submicroscopic *P*. *vivax* was associated with anemia^16^, but this association was not seen in our studies, or in a study in Indonesia^13^.

In our community surveys, age was a risk factor for malaria mainly in Rourkela, the site with the highest prevalence of *P*. *falciparum*; compared to adults, young children (<5 years) were significantly more likely to have microscopic *P*. *falciparum* and microscopic and submicroscopic *P*. *vivax*, whereas older children (5–14 years) were more at risk for *P*. *falciparum*. In areas with multiple malaria parasite species, it has been suggested that the acquisition of immunity to *P*. *vivax* develops faster, with a peak of *P*. *vivax* at younger ages compared to *P*. *falciparum*, and an earlier ability to keep parasite densities of *P*. *vivax* infections under control^17,18^. Age patterns similar to our observations have been described in studies from Bangladesh, Papua New Guinea, Thailand, and the Amazon in Brazil^16,19–21^. The reasons for the faster acquisition of clinical immunity to *P*. *vivax* are not well understood, but repeated exposures from hypnozoites might be a contributing factor^22,23^.

Males were at higher risk of malaria at all of our sites. The difference in malaria risk by gender among adults has been reported before in Asia^15,24^ and Ethiopia^25^, and has been attributed to more outdoor activity by men in areas, such as forests, with infected mosquitoes. However, the increased risk was also observed in our site in the urban city of Chennai that has few forested areas. This higher risk might be due to different use of malaria prevention methods, distinctive attractiveness to mosquitoes, different travel patterns by gender, or hormonal or host-genetic factors^24^. A better understanding of the factors contributing to the gender gap may assist in identifying more efficient ways to prevent malaria among males, by determining, e.g., if the increased risk among males is exposure-related on account of different habits before bed time compared to women.

Other risk factors for malaria included travel for *P*. *vivax* in the community surveys in Chennai and Nadiad, perhaps indicating that not all malaria detected in these locations originated at those sites. Although ITNs were only used in Rourkela, they were more a marker of malaria risk than associated with protection, as has been previously reported^26^, whereas the use of repellents was associated with reduced malaria risk in Nadiad and Rourkela.

At our site in Nadiad, almost all infections in the clinic study and community survey were microscopic. Nadiad has a history of low malaria prevalence, so residents can be expected to have low immunity, which may explain why most infections will develop parasitaemias detectable by microscopy. However, even in this area with sustained low transmission, one out of five malaria infections identified in the community surveys was asymptomatic. A review of submicroscopic *P*. *falciparum* infections suggested that submicroscopic carriers actually predominate in areas of lower transmission, which would accord with our observations at our other sites^27^.

Several factors have been associated with submicroscopic and asymptomatic *Plasmodium* infections. Recent use of an antimalarial drug can reduce *Plasmodium* infections to submicroscopic levels as part of the normal process of clearing parasites. However, persistence of submicroscopic levels can occur after treatment when drug resistance is present or when inadequate doses of antimalarials are used. In areas with both *P*. *vivax* and *P*. *falciparum*, treatment of *P*. *falciparum* or mixed species infections has been associated with an increased risk of the occurrence of *P*. *vivax*^28^. Recent antimalarial use was associated with symptomatic and asymptomatic *P*. *vivax* infections in community studies in Nadiad and symptomatic *P*. *vivax* in community studies in Rourkela, and with submicroscopic malaria in clinic studies in Chennai. *P*. *falciparum* resistance to chloroquine is well known, but *P*. *vivax* resistance to chloroquine has been infrequently reported in India^29,30^; currently the combination artesunate-sulfadoxine-pyrimethamine is used with good reported efficacy in our study sites^31^.

Although it has been suggested that mixed species infections may be more commonly detected by PCR compared to microscopy^32^, we did not find a large discrepancy in number detected by the two methods (Supplemental Table S1). While mixed species infections were more common than would be expected from the prevalence of single infections in our community surveys, their overall prevalence was low, in accord with other reports from surveys in Asia (Supplemental Table S5). It has been suggested that one species may suppress another, or that multiple species in a region may attenuate clinical disease^33^. Mixed infections were too rare in our study to support meaningful analysis, but patterns of malaria prevalence by age, gender, and species in Rourkela indirectly show that immunity development is different for both species (Supplemental Table S1).

Our spatial data analysis demonstrated how local spatial autocorrelation techniques on high resolution (individually georeferenced) datasets can be used to identify specific areas of interest (AOIs) in which there are unexpectedly high concentrations of *Plasmodium* infections. Though there has been a great deal of interest in the application of autocorrelation and other spatial analysis techniques (‘spatial epidemiology’^34^) in the literature in recent years, leading to substantial improvements in risk mapping for malaria and other diseases such as dengue^8,35,36^, this work has almost exclusively been undertaken using historical data that have been aggregated to the district or village level (e.g.^7,8,34^). In contrast to this, our analysis of individually georeferenced PCR results allowed analysis to take place without the generalisation and aggregation that are typically exhibited in datasets relating to disease prevalence. Accordingly, specific AOIs have been identified for further analysis, rather than simply the district within which hotspots are contained. Once identified, AOIs can undergo further scrutiny either statistically, using techniques such as Geographically Weighted Regression in order to examine the relationship between hotspots and social, entomological, or environmental factors; or in the field, by visiting the locality in order to identify behaviours or factors that may be contributing to these hotspots, and propose interventions in order to reduce risk. Additional collection of temporal data too will enable changes in risk factors, malaria incidence, and the resulting autocorrelation results over time to be discerned, as reported by other studies^35,36^.

Our study had several limitations. First, factors which can contribute to the detection of submicroscopic infections include the skill of the microscopist, the quality of the PCR reaction, the amount of genetic material used for PCR, and host and parasite factors^4^. The microscopists at our sites were trained and experienced, and the PCR tests were were performed by experienced molecular biology technicians at each site and repeated at least twice with cross-checking of a subset in a second lab. Parasite genetic diversity may affect submicroscopic infections and this is still under study at our sites, and associations between other host genetic factors such as hemoglobinopathies were not examined. We did not determine if persons identified with asymptomatic infections subsequently developed symptoms, or if they were able to clear the parasites. The surveys in Chennai and Rourkela were underpowered and it is possible that some associations with malaria were not significant because of low numbers. Second, because of the timing of the interviews during the daytime, our surveys did not capture all household members; in Nadiad and Chennai, men and children were underrepresented compared to the census (Supplemental Fig, S1), and this may have lead to an underestimation of the real malaria prevalence if children and men are more at risk of malaria. While logistically challenging, undertaking interviews early in the morning or later in the afternoon and on weekends to capture all household members may be a better approach in future studies.

In summary, although the overall burden of malaria at our three sites in India was low, the contribution of submicroscopic infections (range 3–74%) and of asymptomatic infections (range 18–74%) should not be underestimated in light of malaria elimination efforts. This is especially true for *P*. *vivax*, where hypnozoites, a source of recurrent infections, are unlikely to be treated if the infection is not identified. In addition, the gametocytes of *P*. *vivax* in asymptomatic infections have been shown to be as infectious as in symptomatic malaria^37^ and *P*. *falciparum* gametocytes can be as common or more prevalent in asymptomatic compared to symptomatic infections^38^. In malaria clinics, submicroscopic infections are of concern because patients may not receive the treatment they need. Since molecular methods such as PCR are not suitable for routine use as a diagnostic in a health care setting due to the costs, training, and equipment involved, ultra-sensitive point-of-care tools are urgently needed^39^.

## Methods

### Study sites and population

Chennai city, the capital of the southern state of Tamil Nadu and the sixth most populous city with ~7 million people, is located on the coast of the Bay of Bengal. The main rainfall period is from October-December as part of the northeast monsoon, but rains also fall during the southwest monsoon between July-August^40^. Malaria transmission (predominantly *P*. *vivax*) in Chennai is perennial and peaks between July-October. The Besant Nagar Malaria Clinic is in a predominantly residential neighbourhood composed of middle- and upper-class dwellings, along with a few slums and a large coastal fishing community. Subjects were enrolled from the Besant Nagar Malaria Clinic and the surrounding Besant Nagar catchment area. Nadiad city, population ~225,000, is located in the district of Kheda in the central part of Gujarat State. Nadiad receives the majority of its rain during the southwest monsoon season (June-September)^40^. Malaria is considered to be unstable (hypo-endemic), with *P*. *vivax* and *P*. *falciparum* prevalence rates oscillating throughout the year based on the rainfall. The National Institute of Malaria Research (NIMR) Malaria Clinic is located in the Civil Hospital of Nadiad in a predominantly residential neighbourhood. Subjects were enrolled at the Civil Hospital outpatient clinic, and community surveys were conducted in areas adjacent to Nadiad town. Rourkela city, population ~483,000, is located in the Sundargarh district of the eastern state of Odisha. Rourkela receives rains during the southwest monsoon season (June-September) and some rainfall during the northeast monsoon (December-January)^40^. Historically, malaria transmission has been meso- to hyper-endemic in this region, with *P*. *falciparum* as the major infecting species, accounting for >40% of the total cases in the country. Subjects were enrolled from a malaria clinic at Sector 1 in Rourkela, whereas community surveys were conducted in areas close to forested regions around Rourkela.

### Study designs

A clinic study and community surveys were conducted in each setting. For the clinic studies, symptomatic subjects aged 12 months to 69 years who self-presented with complaints of illness to the clinic for malaria testing were eligible for enrollment, while pregnant women were excluded; a convenience sample was used. After consent, individuals underwent a physical examination, were asked questions about their history of malaria, and had finger-prick blood collected for hemoglobin measurement, blood smear, and PCR. Persons with a positive malaria test in the clinic were treated as per national guidelines; treatment was not directly observed (*P*. *vivax*: chloroquine 25 mg/kg over three days and primaquine 0.25 mg/kg for 14 days; *P*. *falciparum* artesunate 4 mg/kg for 3 days in combination with sulfadoxine 25 mg/kg and pyrimethamine 1.25 mg/kg on the first day and primaquine 0.75 mg/kg)^41^. A census was conducted before the start of the community surveys. The community surveys were conducted in random household samples of the census and were conducted over the course of two years spread around the seasons; If a household was not available, the next household on the list was used. All members available in the household were sampled during day time hours of 7AM to 3 PM. We did not return to a household to find missing household members. Compared to the census, children were under-represented in Chennai and Nadiad, whereas women were overpresented in all sites (Supplemental Fig. S1). The latitude and longitude of each household was taken using a handheld GPS device. Procedures and exclusion criteria were the same as for the studies in the clinics. For the surveys, sample sizes were estimated based on a two-tailed, point biserial model with a conservative 95% confidence interval and a power ≥90% (G*Power v3.1.2) for a range of prevalences; for Chennai, the prevalence was expected to be around 12–16% with estimated sample sizes of 459–950, in Nadiad the expected prevalence range was 7–11% with estimated sample sizes of 780–2000, and corresponding numbers in Rourkela were 19–26% and estimated sample size of 109–355. The surveys aimed for 400 participants every 3 months (per site), but because of logistical constraints (availability of staff, poor weather conditions) this was not always feasible.

### Diagnostic methods

Hemoglobin level was assessed using HemoCue (Ängelholm, Sweden). Thin and thick smears were Giemsa stained and at least 300 fields in the thick smear were examined by microscopy using a 100x oil immersion objective. Parasites were counted on the thick smear against 200–500 leucocytes. The results were expressed as parasites per microliter of blood, using the white blood cell (WBC) count if known, or assuming 8,000 WBCs per microliter of blood. Slides were routinely read by two microscopists, and a third microscopist was used if there was disagreement. All samples, whether positive or negative by microscopy, underwent DNA extraction by QIAamp DNA blood Mini Kits (Qiagen Inc., Valencia, CA). A modified nested, multiplex-PCR method targeting the 18 S small subunit ribosomal DNA gene (SSU rDNA) was used for species-specific detection of *Plasmodium* parasites^42,43^. Amplicons were visualized on a 1.5% agarose gel, and fragment sizes determined using a 100 bp DNA ladder (exACT gene, Thermo Fisher Scientific, Pittsburg, PA). All samples were amplified by PCR at least twice, and a third time if any discrepancy, and 10% sent for cross-checking at a central lab facility. Positive controls consisting of known *P*. *falciparum*, *P*. *vivax* and *P*. *malariae* DNA and negative controls (no DNA) were used for every amplification experiment.

### Analysis

A secure, web-based REDCap (Research Electronic Data Capture) database was used to capture and store all subject data and test results^44^, and data were exported into Stata (Stata/IC version 14.2, StataCorp LP, College Station, USA) for analysis. We classified parasite infections detected by PCR but not by microscopy as ‘submicroscopic’. We defined malaria as symptomatic if the subject was *Plasmodium*-positive by PCR and had documented fever (a body temperature of ≥37.5 °C) or a history of fever in the past 48 hours. For anemia, an age- and gender-appropriate definition was used^45^: <11 g/dl if <5 years, <11.5 g/dl if 5–11 years, <12 g/dl if 12–14 years or >15 years and female,<13 g/dl if male and >15 years. In all three study areas, risk factors for submicroscopic and asymptomatic malaria were examined among community survey participants, and for submicroscopic malaria among clinic participants. Risk factors included those known from literature, such as age, gender, a history of travel in the past two weeks, a history of malaria in the past year, antimalarial use in the past two weeks, reported use of repellents (coils, vaporizers), and season at the time of visit. Generalized linear regression with a log link and binomial distribution was used for multivariate analyses, and Poisson regression with a robust variance estimator was used for models which did not converge^46^. Factors with a p-value < 0.15 in the univariate model were included in the multivariate model, and factors with a p-value > 0.08 were removed from the multivariate model. All community survey analyses were weighted by age and gender, using the household information obtained from the census (svy procedure in Stata 14.2). To assess a potential effect of malaria infections on fever and anemia, we combined the data in forest plots using fever and anemia among persons without malaria by PCR as the reference group (random effects model); the *I*^2^ was used as an indicator of heterogeneity between studies.

### Exploratory spatial data analysis

We used Exploratory Spatial Data Analysis (ESDA)^7^ to visualize spatial distributions of a subset of 3,001 subjects enrolled in the community studies at Chennai (n = 907), Rourkela (n = 1306) and Nadiad (n = 788). Local Indicators of Spatial Association (LISA) were calculated using Local Moran’s *I* analysis^47^, operationalized using the PySAL library for Python (https://pysal.readthedocs.io/en/latest/). As with similar spatial analysis techniques (e.g. *Gi* and Gi*^48^), Moran’s *I* may be used to test the null hypothesis of no local spatial association. Pseudo-significance levels were calculated using 9999 random permutations of the analysis. We constructed a ‘spatial weights’ matrix, containing a representation of the neighbourhood structure of the study area and containing all of the points that fall within a certain distance threshold of another point, referred to as the ‘neighbours’ for that point, with each value representing a standardised distance between the points. This approach minimises sensitivity to variations in the distance threshold because closer points are more important than those further away. For the purposes of this analysis, the threshold at each site is calculated as the smallest distance at which each point is ensured at least one neighbour, and the matrix is row standardised (such that all elements in each row sum to one) to facilitate interpretation of the statistics^47^.

### Ethical clearance

Institutional Review Board approval was obtained from New York University, and Institutional Ethical Clearance from NIMR. Informed consent was obtained from all subjects or, if subjects were under 18, from a parent and/or legal guardian. All experiments were performed in accordance with the relevant guidelines and regulations.

### Additional information

Supplementary information accompanies this paper. Data are available through the Clinical Epidemiology Database, ClinEpiD (https://clinepidb.org), part of the EuPathDB project^49^.

## Supplementary information

SUPPLEMENTARY MATERIAL
