# Supplementary material for: The burden of submicroscopic and asymptomatic malaria in India revealed from epidemiology studies at three varied transmission sites in India
Source: Sci Rep. 2019 Nov 19;9:17095. doi: 10.1038/s41598-019-53386-w (PMC6863831; doi:10.1038/s41598-019-53386-w)
Supplement: Supplementary file 1 — SUPPLEMENTARY MATERIAL [file 41598_2019_53386_MOESM1_ESM.docx]

Supplementary material:

**The burden of submicroscopic and asymptomatic malaria in India revealed from epidemiology studies at three varied transmission sites in india**

Anna Maria van Eijk, Patrick L. Sutton, Lalitha Ramanathapuram, Steven A. Sullivan, Deena Kanagaraj, G Sri Lakshmi Priya, Sangamithra Ravishankaran, Aswin Asokan, V. Sangeetha, Pavitra N. Rao, Samuel C. Wassmer, Nikunj Tandel, Ankita Patel, Nisha Desai, Sandhya Choubey, Syed Zeeshan Ali, Punam Barla, Rajashri Rani Oraon, Stuti Mohanty, Shobhna Mishra, Sonal Kale, Nabamita Bandyopadhyay, Prashant K. Mallick, Jonathan Huck, Neena Valecha, Om P. Singh, K Pradhan, Ranvir Singh, S. K. Sharma, Harish C. Srivasatava, Jane M. Carlton, Alex Eapen

Contents

[Table S1. Malaria as detected by microscopy and PCR in community surveys and clinic studies at three study sites in India, 2012-2015. 2](#_Toc20680806)

[Table S2. Factors associated with malaria, by detection method and presence of fever in multivariate analyses, community surveys at three sites in India, 2012-2014. 3](#_Toc20680807)

[Table S3. Comparison of gametocytemia and parasite and gametocyte densities among symptomatic and asymptomatic malaria in the community surveys 6](#_Toc20680808)

[Table S4. Factors associated with microscopic and submicroscopic malaria in clinic studies in three sites in India, 2012-2015 7](#_Toc20680809)

[Table S5. Mixed infections reported in recent studies in Asia and Ethiopia 8](#_Toc20680810)

[Figure S1. Age (A) and gender (B) categories in census and surveys in three sites in India, 2012-2015 9](#_Toc20680811)

[Figure S2. Malaria prevalence diagnosed by PCR by season in surveys and clinic studies in three sites in India, 2012-2015 10](#_Toc20680812)

[Figure S3. The association between anemia and microscopic and submicroscopic malaria in three clinic studies in India, 2012-2015 11](#_Toc20680813)

[Figure S4. The risk of *P. vivax* among persons with and without *P. falciparum* in community surveys and clinic studies in three sites in India, 2012-2015 12](#_Toc20680814)

[References 13](#_Toc20680815)

## Table S1. Malaria as detected by microscopy and PCR in community surveys and clinic studies at three study sites in India, 2012-2015.

|  | **Community surveys**  (weighted by age and gender; absolute number of infections in brackets) | | | **Clinic studies**  (number of infections in brackets) | | |
| --- | --- | --- | --- | --- | --- | --- |
|  | **Chennai** (N=928)  **% (95% CI)** | **Nadiad** (N=796)  **% (95% CI)** | **Rourkela** (N=1307)  **% (95% CI)** | **Chennai** (N=1054)  **% (95% CI)** | **Nadiad** (N=685)  **% (95% CI)** | **Rourkela** (N=1875)  **% (95% CI)** |
| Microscopy: any | 0.8, 0.4-1.6* (8) | 7.9, 6.1-10.1 (58) | 8.2, 6.9-9.7 (105) | 17.6, 15.5-20.1 (186) | 10.4, 8.3-12.9 (71) | 3.4, 2.6-4.3† (63) |
| Pf | 0.2, 0.0-0.9 (1) | 2.1, 1.3-3.4 (16) | 6.6, 5.5-8.0 (85) | 1.5, 0.9-2.5 (16) | 1.5, 0.1-2.7 (10) | 2.1, 1.6-2.9 (40) |
| Pv | 0.6, 0.3-1.3 (7) | 5.5, 4.0-7.5 (40) | 1.1, 0.7-1.8 (14) | 15.8, 13.8-18.2 (167) | 8.9, 7.0-11.3 (61) | 1.1, 0.7-1.6 (20) |
| Pf and Pv | 0 | 0.3, 0.1-1.0 (2) | 0.5, 0.2-1.0 (6) | 0.3, 0.1-0.9 (3) | 0 | 0.1, 0.0-0.4 (2) |
| Pm | 0 | 0 | 0 | 0 | 0 | 0.05, 0.0-0.4 (1) |
| Gametocytes Pf | 0* | 1.4, 0.1-2.5 (11) | 0.6, 0.3-1.2 (7) | 0.6, 0.3-1.3 (0.6) | 1.2, 0.6-2.3 (8) | 0‡ |
| Gametocytes Pv | 0.6, 0.3-1.3 (7) | 5.8, 4.3-7.8 (42) | 0.1, 0.0-0.4† (1) | 15.7, 13.7-18.1 (166) | 8.9, 7.0-11.3 (61) | 0.2, 0.1-0.6† (4) |
| Parasite density: geometric mean/µl, 95% CI (not weighted) § | | | | | | |
| *P. falciparum* | 7000, (1) | 2023, 774-5289, (16) | 1470, 962-2246, (86) | 921, 418-2031,  (19) †† | 1880, 528-6691, (10) | 4109, 2024-8341, (40) |
| *P. vivax* | 1179, 31-45409,  (4) | 1859, 1249-2768,  (42) | 863, 290-2567, (16) | 1592, 1260-2010, (165)* | 2540, 1862-3465, (61) | 4672, 1892-11534, (22) |
| Gametocytes *P. falciparum* | 0 | 699, 406-1204,  (11) | 174, 32-940,  (7) | 709, 78-6425,  (6) | 611, 286-1306,  (8) | 0 |
| Gametocytes *P. vivax* | 198, 51-768,  (7) | 1311, 1015-1695, (42) | 320, (1) | 821, 680-992,  (166) | 1121, 802-1566,  (61) | 1056, 25-45269,  (4) |
| PCR: any | 2.7, 1.8-4.1* (24) | 7.6, 5.8-9.8 (56) | 8.3, 7.0-9.9 (101) | 21.1, 18.7-23.6 (222) | 9.6, 7.6-12.1 (66) | 5.0, 4.1-6.1† (94) |
| Pf | 0.7, 0.3-1.5 (6) | 2.1, 1.3-3.3 (16) | 6.5, 5.3-7.9 (78) | 2.8, 1.9-3.9 (29) | 1.6, 0.9-2.9 (11) | 3.4, 2.6-4.3 (63) |
| Pv | 1.9, 1.1-3.2 (17) | 5.2, 3.8-7.2 (38) | 1.5, 1.0-2.2 (18) | 17.9, 15.7-20.4 (189) | 8.0, 6.2-10.3 (55) | 1.2, 0.8-1.8 (22) |
| Pf and Pv | 0.1, 0.0-0.7 (1) | 0.2, 0.0-0.9 (2) | 0.4, 0.1-0.8 (5) | 0.4, 0.1-1.0 (4) | 0 | 0.2, 0.1-0.6 (4) |
| Pf and Pm | 0 | 0 | 0 | 0 | 0 | 0.3, 0.1-0.6 (5) |
| Pf: Pv ratio | 0.4 | 0.4 | 4.3 | 0.2 | 0.2 | 2.8 |
| PCR vs. microscopy | N=928 | N=796 | N=1307 | N=1054 | N=685 | N=1875 |
| Malaria by PCR & microscopy | 0.7, 0.3-1.5 (7) | 7.3, 5.6-9.5 (54) | 5.7, 4.6-7.0† (70) | 17.1, 14.9-19.5 (180) | 9.5, 7.5-11.9 (65) | 2.7, 2.1-3.6† (51) |
| Pf | 0.1 (1) | 2.0 (15) | 4.5 (56) | 1.5 (16) | 1.5 (10) | 1.7 (31) |
| Pv | 0.6 (6) | 5.1 (37) | 0.9 (10) | 15.5 (163) | 8.0 (55) | 0.9 (16) |
| Pf & Pv | 0 | 0.2 (2) | 0.3 (4) | 0.1 (1) | 0 | 0.2 (4) |
| Submicroscopic malaria | 2.0, 1.2-3.3 (17) | 0.2, 0.1-0.9 (2) | 2.6, 1.9-3.6 (31) | 4.0, 3.0-5.4 (42) | 0.1, 0.0-1.0 (1) | 2.2, 1.7-3.0 (42) |
| Pf | 0.5 (5) | 0.1 (1) | 1.9 (22) | 1.2 (13) | 0.1 (1) | 1.7 (32) |
| Pv | 1.3 (11) | 0.1 (1) | 0.6 (8) | 2.5 (26) | 0 | 0.3 (6) |
| Pf & Pv | 0.1 (1) | 0 | 0.1 (1) | 0.3 (3) | 0 | 0 |
| Pf & Pm | 0 | 0 | 0 | 0 | 0 | 0.2 (4) |
| PCR negative | 97.3, 95.9-98.2 (904) | 92.4, 90.2-94.2 (740) | 91.7, 90.1-93.0 (1206) | 78.9, 76.4-81.3 (832) | 90.4, 87.9-92.4 (619) | 95.0, 94.0-95.9 (1781) |
| Malaria by fever status** | N=919 | N=794 | N=1299 |  |  |  |
| Symptomatic | 0.8, 0.4-1.6 (7) | 6.1, 4.6-8.2 (44) | 2.9, 2.1-3.8† (36) |  |  |  |
| Pf | 0.2 (1) | 1.9 (14) | 2.5 (31) |  |  |  |
| Pv | 0.6 (6) | 4.3 (30) | 0.2 (3) |  |  |  |
| Pf & Pv | 0 | 0 | 0.2 (2) |  |  |  |
| Asymptomatic | 2.0, 1.2-3.3 (17) | 1.4, 0.8-2.5 (12) | 5.5, 4.4-6.8 (65) |  |  |  |
| Pf | 0.6 (5) | 0.2 (2) | 4.0 (47) |  |  |  |
| Pv | 1.3 (11) | 1.0 (8) | 1.3 (15) |  |  |  |
| Pf & Pv | 0.1 (1) | 0.2 (2) | 0.2 (3) |  |  |  |
| None | 97.2, 95.8-98.2 (895) | 92.4, 90.2-94.2 (738) | 91.6, 90.1-93.0 (1198) |  |  |  |

Abbreviations: CI: confidence interval, PCR: polymerase chain reaction, Pf: *Plasmodium falciparum*, Pv: *Plasmodium vivax*, Pm: *Plasmodium malariae*

* P<0.05 comparing Chennai to Nadiad and Rourkela (Chi-square test)

† P<0.05 comparing to each other (Chi-square test or t-test for parasite densities)

‡ P<0.05 comparing Rourkela to Chennai and Nadiad (Chi-square test)

§ *P. falciparum* count missing for 2 participants in the community survey in Nadiad and for 5 participants in the community survey and 2 participants in the clinic study in Rourkela; *P. vivax* count missing for 5 participants in the clinic study in Chennai and 4 in the community survey in Rourkela. Three *P. vivax* infections in the community survey in Chennai only showed gametocytes

** Fever defined as a history of fever in the past 48 hours or documented fever (≥ 37.5 °C), malaria parasites detected by PCR

†† P<0.05, Chennai vs. Rourkela (t-test)

##

## Table S2. Factors associated with malaria, by detection method and presence of fever in multivariate analyses, community surveys at three sites in India, 2012-2014.

|  | ***Any species*** | | | | ***P. falciparum*** | | | | | ***P. vivax*** | | | | |
| --- | --- | --- | --- | --- | --- | --- | --- | --- | --- | --- | --- | --- | --- | --- |
|  | **APR, 95% CI** | **p-value** | **APR, 95% CI** | **p-value** | **APR, 95% CI** | **p-value** | | **APR, 95% CI** | **p-value** | **APR, 95% CI** | | **p-value** | **APR, 95% CI** | **p-value** |
| **Chennai survey PCR** | **Malaria by PCR** | |  | | ***Pf* by PCR** | | |  | | ***Pv* by PCR** | | |  | |
| Male |  |  |  |  | 10.31, 1.43-74.54 | | **0.021** |  |  |  | |  |  |  |
| Travel last 2 weeks |  |  |  |  |  | |  |  |  | 2.54, 0.90-7.18 | | 0.078 |  |  |
| Rainy season | 3.57, 1.34-9.53 | **0.011** |  |  |  | |  |  |  | 2.88, 0.94-8.85 | | 0.064 |  |  |
| **Chennai survey** | **Microscopic malaria** | | **Submicroscopic malaria vs. no malaria (PCR-)*** | | **Microscopic *Pf*** | | | **Submicroscopic *Pf* vs. no malaria (PCR-)*** | | **Microscopic *Pv*** | | | **Submicroscopic *Pv* vs. no malaria (PCR-)*** | |
| Age (years) |  |  |  |  |  |  | |  |  |  | |  |  |  |
| <5 |  |  |  |  |  |  | |  |  |  | |  | 5.45, 0.84-35.41 | 0.075 |
| 5-14 |  |  |  |  |  |  | |  |  |  | |  | 2.47, 0.62-9.83 | 0.198 |
| 15+ |  |  |  |  |  |  | |  |  |  | |  | Reference |  |
| Male |  |  |  |  |  |  | | 8.22, 1.11-61.12 | **0.039** |  | |  |  |  |
| Travel last 2 weeks |  |  | 2.52, 0.89-7.11 | 0.081 |  |  | |  |  |  | |  | 3.04, 0.88-10.50 | 0.078 |
| Rainy Season | 7.40, 1.03-53.01 | **0.046** |  |  |  |  | |  |  | 5.85, 0.80-42.78 | | 0.082 |  |  |
| **Chennai survey** | **Symptomatic malaria (PCR)** | | **Asymptomatic malaria vs. no malaria (PCR)*** | | **Symptomatic *Pf* (PCR)** | | | **Asymptomatic *Pf* (PCR) vs. no malaria*** | | **Symptomatic *Pv* (PCR)** | | | **Asymptomatic *Pv* vs. no malaria (PCR)*** | |
| Age (years) |  |  |  |  |  |  | |  |  |  |  | |  |  |
| <5 |  |  |  |  |  |  | |  |  |  |  | | 5.56, 0.85-36.48 | 0.074 |
| 5-14 |  |  |  |  |  |  | |  |  |  |  | | 2.24, 0.54-9.34 | 0.266 |
| 15+ |  |  |  |  |  |  | |  |  |  |  | | Reference |  |
| Male |  |  |  |  |  |  | | 8.14, 1.10-60.45 | **0.040** |  |  | |  |  |
| Travel last 2 weeks | 6.51, 1.50-28.24 | **0.012** |  |  |  |  | |  |  | 11.96, 2.63-54.36 | **0.001** | |  |  |
| Rainy season | 6.27, 0.83-47.3 | 0.075 | 2.98, 0.97-9.20 | 0.058 |  |  | |  |  |  |  | |  |  |
| **Nadiad survey PCR** | **Malaria by PCR** |  |  |  | ***Pf* by PCR** |  | |  |  | ***Pv* by PCR** |  | |  |  |
| Male | 2.09, 1.21-3.59 | **0.008** |  |  | 2.33, 0.91-5.99 | 0.079 | |  |  |  |  | |  |  |
| Antimalarial last 2 weeks | 4.66, 2.90-7.49 | **<0.001** |  |  |  |  | |  |  |  |  | |  |  |
| Travel last 2 weeks |  |  |  |  |  |  | |  |  | 2.54, 0.90-7.18 | 0.078 | |  |  |
| Uses repellents | 0.47, 0.26-0.86 | **0.015** |  |  |  |  | |  |  |  |  | |  |  |
| Rainy season | 2.65, 1.50-4.63 | **0.001** |  |  | 0.39, 0.14-1.05 | 0.062 | |  |  | 2.88, 0.94-8.85 | 0.064 | |  |  |
| **Nadiad survey** | **Symptomatic malaria (PCR)** | | **Asymptomatic malaria vs. no malaria (PCR)*** | | **Symptomatic *Pf* (PCR)** | | | **Asymptomatic *Pf* (PCR) vs. no malaria*** | | **Symptomatic *Pv* (PCR)** | | | **Asymptomatic *Pv* vs. no malaria (PCR)*** | |
| Age (years) |  |  |  |  |  |  | |  |  |  | |  |  |  |
| <5 |  |  |  |  |  |  | |  |  | 3.94, 1.11-13.99 | | **0.034** |  |  |
| 5-14 |  |  |  |  |  |  | |  |  | 1.29, 0.58-2.89 | | 0.535 |  |  |
| 15+ |  |  |  |  |  |  | |  |  | Reference | |  |  |  |
| Male | 2.77, 1.41-5.43 | **0.003** |  |  | 4.89, 1.42-16.81 | **0.012** | |  |  | 2.36, 1.04-5.36 | | **0.040** |  |  |
| Travel last 2 weeks |  |  | 3.70, 1.29-10.62 | **0.015** |  |  | |  |  |  | |  | 4.99, 1.66-14.97 | **0.004** |
| Uses repellents |  |  |  |  |  |  | |  |  | 0.35, 0.14-0.85 | | **0.021** |  |  |
| Antimalarial last 2 weeks | 5.77, 3.29-10.13 | **<0.001** | 9.11, 3.18-26.05 | **<0.001** |  |  | | 8.42, 0.97-73.0 | 0.053 | 7.34, 3.86-13.94 | | **<0.001** | 7.48, 2.36-23.72 | **0.001** |
| Rainy season | 2.39, 1.33-4.29 | **0.004** |  |  | 0.31, 0.09-1.07 | 0.065 | |  |  | 10.57, 3.39-32.96 | | **<0.001** |  |  |
| **Rourkela survey PCR** | **Malaria by PCR** | |  | | ***Pf* by PCR** | | |  | | ***Pv* by PCR** | | |  | |
| Age (years) |  |  |  |  |  |  | |  |  |  | |  |  |  |
| <5 | 1.42, 0.82-2.47 | 0.210 |  |  | 1.45, 0.79-2.66 | 0.229 | |  |  | 3.08, 1.20-7.93 | | **0.020** |  |  |
| 5-14 | 1.89, 1.32-2.70 | **0.001** |  |  | 2.03, 1.37-3.01 | **<0.001** | |  |  | 1.91, 0.81-4.47 | | 0.137 |  |  |
| 15+ | Reference |  |  |  | Reference |  | |  |  | Reference | |  |  |  |
| Male | 1.95, 1.37-2.77 | **<0.001** |  |  | 1.88, 1.27-2.77 | **0.002** | |  |  | 2.60, 1.17-5.82 | | **0.020** |  |  |
| ITN use | 1.94, 1.38-2.72 | **<0.001** |  |  | 2.45, 1.66-3.61 | **<0.001** | |  |  |  | |  |  |  |
| Use of repellents |  |  |  |  |  |  | |  |  | 0.29, 0.10-0.86 | | **0.026** |  |  |
| Rainy season | 2.64, 1.80-3.85 | **<0.001** |  |  | 3.31, 2.09-5.22 | **<0.001** | |  |  |  | |  |  |  |
| **Rourkela survey** | **Microscopic malaria** | | **Submicroscopic malaria vs. no malaria (PCR-)*** | | **Microscopic *Pf*** | | | **Submicroscopic *Pf* vs. no malaria (PCR-)*** | | **Microscopic *Pv*** | | | **Submicroscopic *Pv* vs. no malaria (PCR-)*** | |
| Age (years) |  |  |  |  |  |  | |  |  |  | |  |  |  |
| <5 | 2.29, 1.44-3.63 | **<0.001** |  |  | 2.50, 1.52-4.09 | **<0.001** | |  |  | 3.01, 1.16-7.85 | | **0.024** | 5.74, 1.41-23.28 | **0.015** |
| 5-14 | 2.19, 1.51-3.18 | **<0.001** |  |  | 2.44, 1.64-3.63 | **<0.001** | |  |  | 1.58, 0.60-4.17 | | 0.350 | 2.77, 0.82-9.34 | 0.100 |
| 15+ | Reference |  |  |  | Reference |  | |  |  | Reference | |  | Reference |  |
| Male | 1.60, 1.14-2.24 | **0.007** | 2.45, 1.25-4.82 | **0.009** | 1.39, 0.97-1.98 | 0.070 | | 4.50, 1.85-10.95 | **0.001** | 6.93, 2.26-21.25 | | **0.001** |  |  |
| Past year malaria | 1.40, 1.00-1.98 | 0.053 |  |  | 1.45, 1.01-2.09 | **0.045** | |  |  |  | |  |  |  |
| Antimalarial last 2 weeks |  |  | 2.85, 0.87-9.30 | 0.082 |  |  | |  |  |  | |  | 6.06, 0.87-42.34 | 0.069 |
| ITN use | 1.95, 1.39-2.75 | **<0.001** |  |  | 2.40, 1.64-3.49 | **<0.001** | | 3.92, 1.85-8.30 | **<0.0001** |  | |  |  |  |
| Use of repellents |  |  |  |  |  |  | |  |  |  | |  | 0.19, 0.03-1.21 | 0.078 |
| Rainy season | 1.51, 1.06-2.15 | **0.023** | 38.28, 6.46-227 | <0.001 | 1.76, 1.18-2.62 | **0.006** | | 4.3% vs. 0% dry | **<0.001†** |  | |  | 7.64, 1.95-29.99 | **0.004** |
| **Rourkela survey** | **Symptomatic malaria (PCR)** | | **Asymptomatic malaria vs. no malaria (PCR)*** | | **Symptomatic *Pf* (PCR)** | | | **Asymptomatic *Pf* (PCR) vs. no malaria*** | | **Symptomatic *Pv* (PCR)** | | | **Asymptomatic *Pv* vs. no malaria (PCR)*** | |
| Age (years) |  |  |  |  |  |  | |  |  |  | |  |  |  |
| <5 | 1.80, 0.58-5.61 | 0.307 |  |  | 1.19, 0.31-4.57 | 0.803 | |  |  |  | |  | 2.84, 1.00-8.11 | 0.051 |
| 5-14 | 4.47, 2.39-8.36 | **<0.001** |  |  | 5.18, 2.67-10.05 | **<0.001** | |  |  |  | |  | 1.67, 0.62-4.45 | 0.308 |
| 15+ | Reference |  |  |  | Reference |  | |  |  |  | |  | Reference‡ |  |
| Male |  |  | 2.27, 1.45-3.56 | **<0.001** |  |  | | 2.20, 1.33-3.65 | **0.002** |  | |  | 2.59, 1.07-6.26 | **0.035** |
| Antimalarial last 2 weeks | 3.63, 1.39-9.49 | **0.009** |  |  |  |  | |  |  | 12.23, 1.38-108 | | **0.024** |  |  |
| Travel last 2 weeks | 3.60, 1.02-12.72 | **0.047** |  |  | 4.28, 1.23-14.90 | **0.022** | |  |  |  | |  |  |  |
| ITN use | 2.36, 1.27-4.36 | **0.006** | 1.78, 1.17-2.70 | **0.008** | 2.41, 1.27-4.56 | **0.007** | | 2.48, 1.50-4.11 | **<0.001** | 7.80, 0.82-74.35 | | 0.074 |  |  |
| Use of repellents |  |  |  |  |  |  | |  |  |  | |  | 0.09, 0.02-0.56 | **0.010** |
| Rainy season | 2.43, 1.16-5.08 | **0.018** | 2.74, 1.71-4.39 | **<0.001** | 2.36, 1.17-4.76 | **0.002** | | 4.50, 2.39-8.50 | **<0.001** | 0.9% vs. 0% dry | | **0.016**† |  |  |

Abbreviations: APR: adjusted prevalence ratio, CI: confidence interval, PCR: polymerase chain reaction, *Pf*: *Plasmodium falciparum*, *Pv*: *Plasmodium vivax*

Note: All models weighted for age and gender using the census information. Note that sample sizes for some outcomes were small; we presented all results with a p-value <0.08 in this table, to allow the readers to evaluate variables which had a “borderline significant” association with malaria.

*Reference group: no malaria by PCR

†Fisher’s exact test

‡ For asymptomatic *P. vivax* infections: Age <15 vs. 15+: prevalence ratio 2.08, 95% CI 0.90-4,82, p=0.088

## Table S3. Comparison of gametocytemia and parasite and gametocyte densities among symptomatic and asymptomatic malaria in the community surveys

|  | **Chennai**  **Gametocytemia** | | **Nadiad**  **Gametocytemia** | | | **Rourkela**  **Gametocytemia** | | |
| --- | --- | --- | --- | --- | --- | --- | --- | --- |
| (Gametocytes by microscopy) | Symptomatic malaria by microscopy (%) | Asymptomatic malaria by microscopy (%) | | Symptomatic malaria by microscopy (%) | Asymptomatic malaria by microscopy (%) | | Symptomatic malaria by microscopy (%) | Asymptomatic malaria by microscopy (%) |
| *P. falciparum* gametocytes | 0/1 Pf infection | 0/0 Pf infection | | 8/13 Pf infections (61.5) | 3/5 Pf infections (60.0) | | 2/37 Pf infections (5.4) | 5/54 Pf infections (9.3) |
| *P. vivax* gametocytes | 4/4 Pv infections (100) | 3/3 Pv infections (100) | | 32/32 Pv infections (100) | 10/10 Pv infections (100) | | 1/4 Pv infections (25.0) | 0/16 Pv infections (0.0) |
|  | Symptomatic malaria by PCR (%) | Asymptomatic malaria by PCR (%) | | Symptomatic malaria by PCR (%) | Asymptomatic malaria by PCR (%) | | Symptomatic malaria by PCR (%) | Asymptomatic malaria by PCR (%) |
| *P. falciparum* gametocytes | 0/1 Pf infections  (0.0) | 0/6 Pf infections  (0.0) | | 8/14 Pf infections (57.1) | 3/4 Pf infections (75.0) | | 2/33 Pf infections (6.1) | 4/50 Pf infections  (8.0) |
| *P. vivax* gametocytes | 4/6 Pv infections (66.7) | 2/12 Pv infections (16.7) | | 29/30 Pv infections (96.7) | 9/10 Pv infections (90.0) | | 1/5 Pv infections (20.0) | 0/18 Pv infections (0.0) |
|  |  |  | | **Density/µl,**  **Geometric mean, 95% CI** | | | **Density/µl,**  **Geometric mean, 95% CI** | |
| *P. falciparum*  Asexual |  |  | | 3214, 906-11402, n=11 | 899, 119-6789,  n=4 | | 4199, 1875-9406, n=31 | 956, 466-1962,  n=28* |
| *P. vivax* asexual |  |  | | 2154, 1439-3224, n=30 | 1751, 438-6995,  n=9 | | 6197  n=2 | 859, 287-2577,  n=9 |
| *P. falciparum* gametocytes |  |  | | 640, 299-1369,  n=8 | 884, 242-3231,  n=3 | | 89  n=2 | 295, 7-11687,  n=4 |
| *P. vivax* gametocytes |  |  | | 1394, 1020-1905, n=30 | 1254, 624-2518,  n=9 | | 320  n=1 | No data |

Pv: *P. vivax*, Pf: *P. falciparum*, CI: confidence interval, µl: microliter

Note: Unweighted data. Only in Nadiad and Rourkela was a sufficient sample size present for a comparison of parasite densities.

*P=0.007 t-test log transformed values comparing parasite densities among symptomatic and asymptomatic *P. falciparum* infections in Rourkela

## Table S4. Factors associated with microscopic and submicroscopic malaria in clinic studies in three sites in India, 2012-2015

|  | **Any species** | | | | ***P. falciparum*** | | | | ***P. vivax*** | | | |
| --- | --- | --- | --- | --- | --- | --- | --- | --- | --- | --- | --- | --- |
|  | **APR, 95% CI** | **p-value** | **APR, 95% CI** | **p-value** | **APR, 95% CI** | **p-value** | **APR, 95% CI** | **p-value** | **APR, 95% CI** | **p-value** | **APR, 95% CI** | **p-value** |
| **Chennai clinic study** | **Malaria by PCR** | |  | | ***P. falciparum* by PCR** | |  | | ***P. vivax* by PCR** | |  | |
| Age <15 years* | 0.33, 0.14-0.79 | **0.012** |  |  |  |  |  |  | 0.30, 0.12-0.80 | **0.016** |  |  |
| Male | 1.36, 1.04-1.78 | **0.027** |  |  |  |  |  |  | 1.37, 1.02-1.84 | **0.035** |  |  |
| Past year malaria | 1.43, 1.10-1.86 | **0.008** |  |  | 2.18, 1.06-4.51 | **0.035** |  |  | 1.37, 1.02-1.83 | **0.039** |  |  |
| Antimalarial last 2 weeks | 1.68, 1.00-2.83 | 0.052 |  |  |  |  |  |  |  |  |  |  |
| Uses repellents | 0.73, 0.56-0.95 | **0.020** |  |  |  |  |  |  | 0.73, 0.55-0.97 | **0.031** |  |  |
| Rainy season | 1.90, 1.44-2.52 | **<0.001** |  |  |  |  |  |  | 2.05, 1.50-2.79 | **<0.001** |  |  |
| **Chennai clinic study** | **Microscopic malaria** | | **Submicroscopic malaria** | | **Microscopic *P. falciparum*** | | **Submicroscopic *P. falciparum*** | | **Microscopic *P. vivax*** | | **Submicroscopic *P. vivax*** | |
| Age <15 years* | 0.40, 0.17-0.95 | **0.038** |  |  |  |  |  |  | 0.37, 0.14-0.97 | **0.044** |  |  |
| Male | 1.57, 1.14-2.17 | **0.006** |  |  |  |  |  |  | 1.64, 1.16-2.31 | **0.005** |  |  |
| Past year malaria | 1.35, 0.99-1.84 | 0.058 | 2.06, 1.08-3.95 | **0.029** |  |  | 2.77, 1.04-7.38 | **0.042** | 1.36, 0.98-1.89 | 0.069 |  |  |
| Antimalarial last 2 weeks |  |  | 3.02, 1.03-8.84 | **0.044** |  |  |  |  |  |  |  |  |
| Uses repellents | 0.72, 0.054-0.97 | **0.032** |  |  |  |  |  |  | 0.73, 0.53-0.99 | **0.045** |  |  |
| Rainy season | 2.11, 1.53-2.91 | **<0.001** | 1.90, 0.99-3.65 | 0.053 |  |  |  |  | 2.26, 1.59-3.21 | **<0.001** |  |  |
| **Nadiad clinic study†** | **Malaria by PCR** | |  | | ***P. falciparum* by PCR** | |  | | ***P. vivax* by PCR** | |  | |
| Male | 2.34, 1.33-4.14 | **0.003** |  |  | 7.12, 0.89-56.97 | 0.064 |  |  | 2.03, 1.11-3.69 | **0.021** |  |  |
| Past year malaria | 2.20, 1.21-4.00 | **0.009** |  |  |  |  |  |  | 2.47, 1.29-4.71 | **0.006** |  |  |
| Antimalarial last 2 weeks |  |  |  |  | 18.41, 6.60-51.34 | **<0.001** |  |  |  |  |  |  |
| Uses repellents |  |  |  |  | 0.23, 0.05-1.06 | 0.059 |  |  |  |  |  |  |
| Rainy season | 1.79, 1.11-2.89 | **0.016** |  |  |  |  |  |  | 1.89, 1.12-3.19 | **0.018** |  |  |
| **Rourkela clinic study** | **Malaria by PCR** | |  | | ***P. falciparum* by PCR** | |  | | ***P. vivax* by PCR** | |  | |
| Male | 1.54, 1.01-2.34 | **0.046** |  |  | 1.79, 1.09-2.95 | **0.022** |  |  |  |  |  |  |
| Past year malaria | 3.74, 2.25-6.20 | **<0.001** |  |  | 2.14, 1.12-4.08 | **0.021** |  |  | 4.76, 2.05-11.06 | **<0.001** |  |  |
| Travel last 2 weeks | 1.70, 1.13-2.55 | **0.011** |  |  | 1.68, 1.04-2.69 | **0.032** |  |  |  |  |  |  |
| Antimalarial last 2 weeks | 0.27, 0.06-1.10 | 0.067 |  |  |  |  |  |  |  |  |  |  |
| Uses repellents |  |  |  |  |  |  |  |  | 0.49, 0.22-1.06 | 0.070 |  |  |
| Rainy season |  |  |  |  |  |  |  |  | 0.33, 0.14-0.77 | **0.011** |  |  |
| **Rourkela clinic study** | **Microscopic malaria** | | **Submicroscopic malaria** | | **Microscopic *P. falciparum*** | | **Submicroscopic *P. falciparum*** | | **Microscopic *P. vivax*** | | **Submicroscopic *P. vivax*** | |
| Male | 1.96, 1.08-3.57 | **0.028** |  |  | 2.82, 1.24-6.44 | **0.014** |  |  |  |  |  |  |
| Past year malaria | 4.14, 2.27-7.56 | **<0.001** |  |  | 2.73, 1.15-6.46 | **0.022** |  |  | 9.42, 3.68-24.12 | **<0.001** |  |  |
| Travel last 2 weeks | 2.22, 1.29-3.82 | **0.004** |  |  | 2.53, 1.30-4.94 | **0.007** |  |  |  |  |  |  |
| Uses repellents | 0.51, 0.30-0.88 | **0.016** |  |  |  |  |  |  |  |  |  |  |
| Rainy season |  |  |  |  |  |  |  |  | 0.19, 0.06-0.66 | **0.009** |  |  |

*Submicroscopic malaria (any species) in Chennai: 0/86 among children <15 years vs. 42/788 (5.3%) among adults, p=0.028, Fisher’s exact test

† *P. falciparum* by PCR in Nadiad: 0/184 among children <15 years vs. 11/501 (2.2%) among adults, p=0.042, Fisher’s exact test

## Table S5. Mixed infections reported in recent studies in Asia and Ethiopia

| Study | Country | Design | Study population | Test | *P. falciparum*  n/N (%) | *P. vivax*  n/N (%) | Mixed  n/N (%) | Risk ratio, 95% CI* | *P* (Fisher exact test) |
| --- | --- | --- | --- | --- | --- | --- | --- | --- | --- |
| Haji 2015^1^ | Ethiopia | Clinic Study | < 16 years | Microscopy | 70/830 (8.4) | 97/830 (11.7) | 3/830 (0.4) | 0.34, 0.11-1.07 | 0.06 |
| Baum 2015^2^ | Thailand | Clinic study | >15 years | qPCR | 7/61 (11.5) | 13/61 (21.3) | 7/61 (11.5) | 1.81, 0.90-3.64 | 0.218 |
| Shahzadi 2013^3^ | Pakistan | Clinic study | Not reported | PCR | 15/100 (15.0) | 16/100 (16.0) | 10/100 (10.0) | 1.88, 0.98-3.58 | 0.120 |
| Golassa 2015^4^ | Ethiopia | Survey | >= 2 years | Microscopy | 25/1094 (2.3) | 22/1094 (2.0) | 8/1094 (0.7) | 11.4, 5.6-23.05 | <0.001 |
| Tripura 2016^5^† | Cambodia | Surveys | all age groups | uPCR | 32/1447 (2.2) | 48/1447 (3.3) | 4/1447 (0.3) | 3.60, 1.37-9.44 | 0.055 |
| Steenkeste 2010^6^ | Cambodia | Survey | All age groups | RFLP-dHPLC | 779/1319 (59.1) | 191/1319 (14.5) | 114/1319 (8.6) | 1.03, 0.79-1.34 | 0.915 |
| Imwong 2015^7^† | Cambodia | Survey | ≥6 months | HVUSqPCR | 32/1447 (2.2) | 48/1447 (3.3) | 4/1447 (0.3) | 2.93, 1.12-7.69 | 0.104 |
| Baum 2015^2^ | Thailand | Survey | > 10 years | qPCR | 8/219 (3.7) | 17/219 (7.7) | 0 | 0-value in cell |  |
| Nguitragool 2017^8^ | Thailand | Survey | All age groups | qPCR | 37/4309 (0.9) | 133/4309 (3.1) | 11/4309 (0.3) | 7.34, 4.26-12.66 | <0.001 |
| Imwong 2015^7^† | Thailand | Survey | ≥6 months | HVUSqPCR | 45/1992 (2.3) | 79/1992 (4.0) | 31/1992 (1.6) | 9.46, 6.69-13.37 | <0.001 |
| Imwong 2015^7^† | Vietnam | Survey | ≥6 months | HVUSqPCR | 87/1536 (5.7) | 230/1536 (15.0) | 21/1536 (1.4) | 1.05, 0.71-1.57 | 0.884 |
| Lover 2018^9^ | Laos | Survey | >18 months | PCR | 5/5802 (0.09) | 28/5802 (0.5) | 6/5802 (0.1) | 129, 67-248 | <0.001 |
| Chaturvedi 2017^10^ | India | Survey | All age groups | Microscopy | 1847/6761 (27.3) | 217/6761 (3.2) | 28/6761 (0.4) | 0.34, 0.23-0.50 | <0.001 |
| Starzengruber 2014^11^ | Bangladesh | Survey | All age groups | PCR | 256/1418 (18.1) | 59/1418 (4.2) | 106/1418 (7.5) | 5.24, 3.90-7.04 | <0.001 |

Abbreviations: HVUSqPCR: high-volume ultrasensitive real-time polymerase chain reaction, PCR: polymerase chain reaction, Pf: *P. falciparum*, Pv: *Plasmodium vivax,* RFLP-dHPLC: Restriction Fragment Length Polymorphism using Denaturing High Performance Liquid Chromatography, qPCR: quantitative PCR, RR: risk ratio, uPCR: ultrasensitive qPCR

* Risk of *P. vivax* among persons with *P. falciparum* compared to persons without *P. falciparum*

†In the study by Tripura *et al.*, species of 142 (9.8%) malaria infections could not be identified^5^. In the study of Imwong *et al*. species of 145 infections (10%) in Cambodia, 182 (12%) in Thailand and 84 (12%) in Vietnam could not be identified^7^. These infections were not included in the calculations of the risk ratio.

## Figure S1. Age (A) and gender (B) categories in census and surveys in three sites in India, 2012-2015

**A**

**B**

## Figure S2. Malaria prevalence diagnosed by PCR by season in surveys and clinic studies in three sites in India, 2012-2015

## Figure S3. The association between anemia and microscopic and submicroscopic malaria in three clinic studies in India, 2012-2015


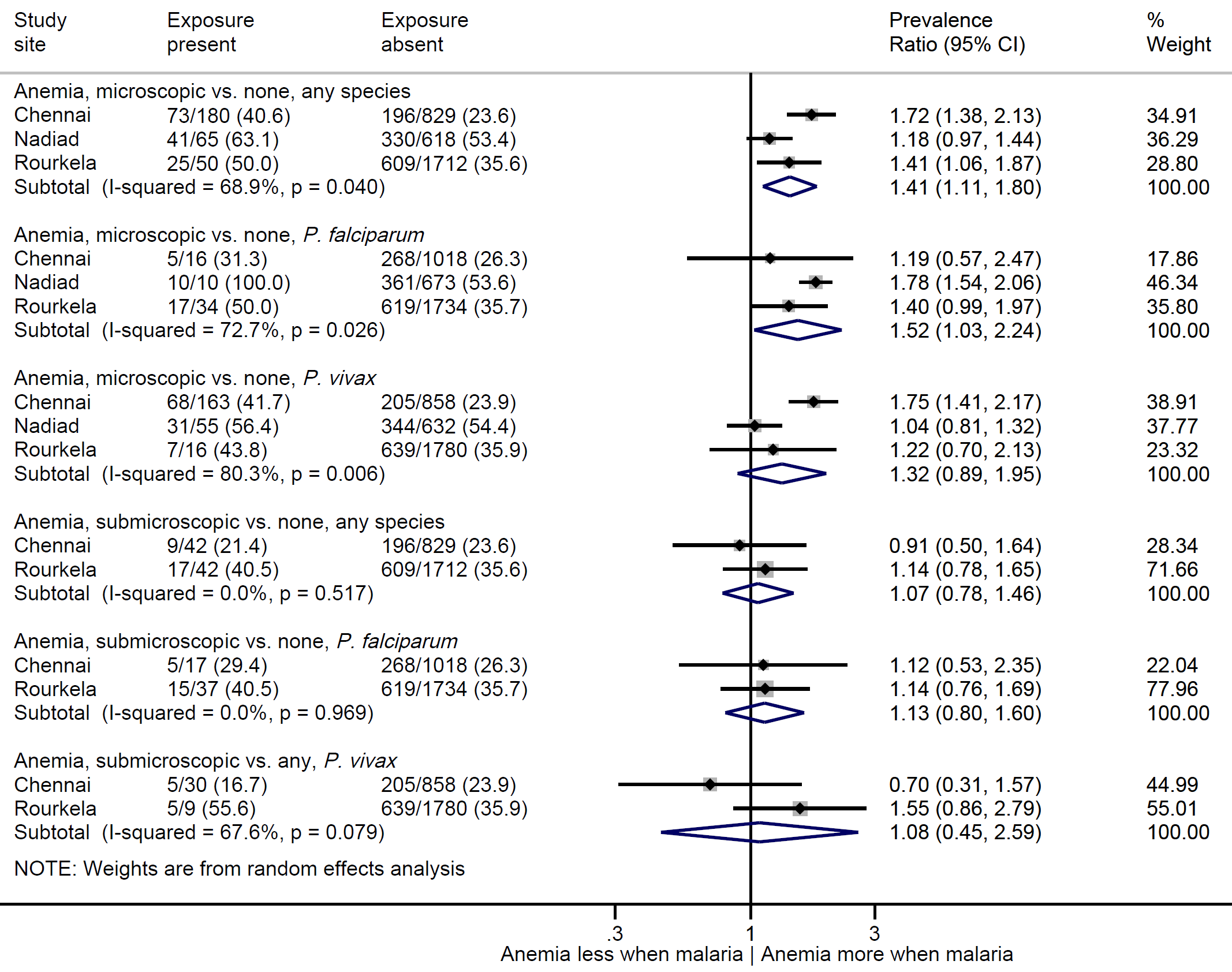


*Only 2 persons with submicroscopic malaria in Nadiad

## Figure S4. The risk of *P. vivax* among persons with and without *P. falciparum* in community surveys and clinic studies in three sites in India, 2012-2015


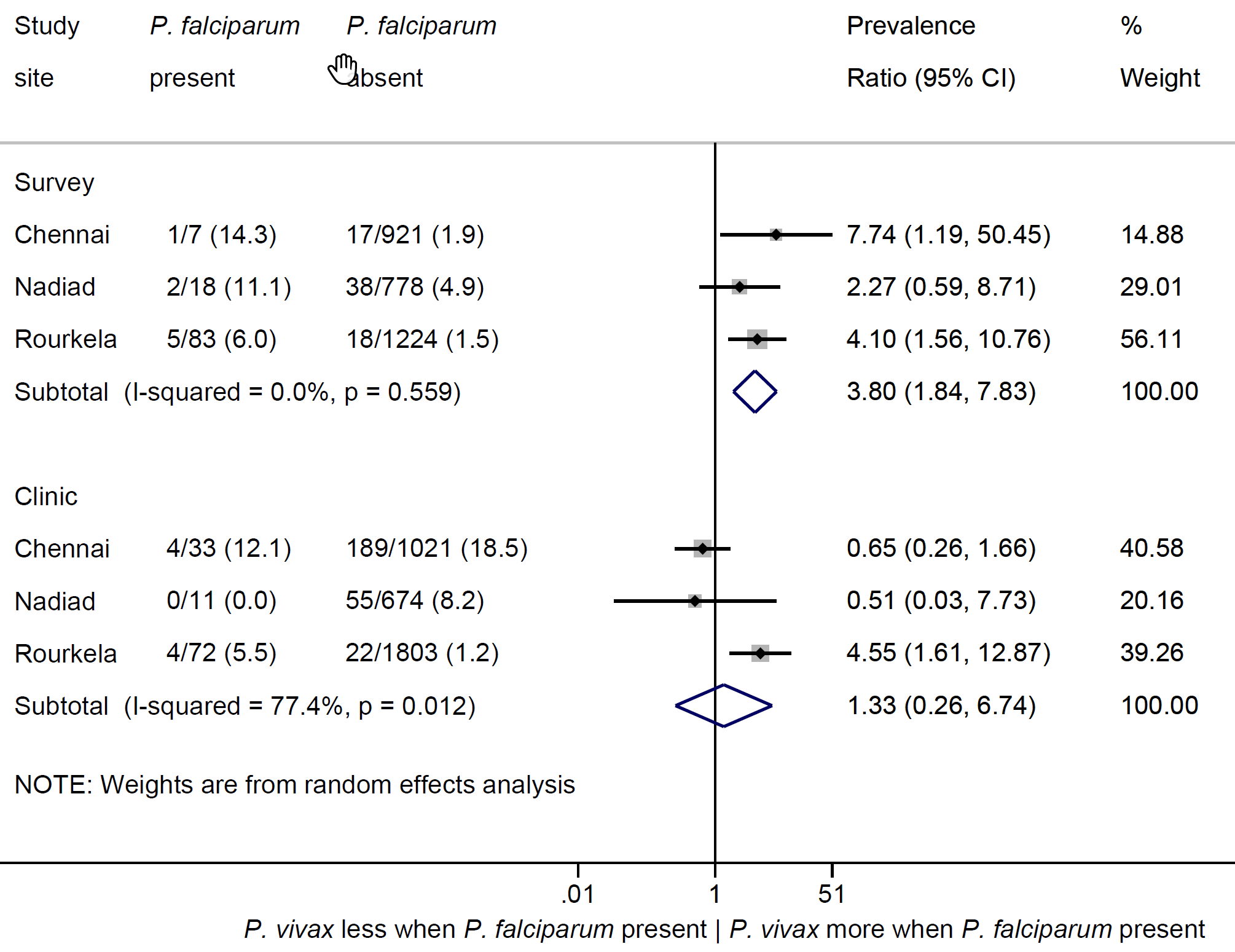


## References

1 Haji, Y., Fogarty, A. W. & Deressa, W. Prevalence and associated factors of malaria among febrile children in Ethiopia: A cross-sectional health facility-based study. *Acta tropica* **155**, 63-70, doi:10.1016/j.actatropica.2015.12.009 (2016).

2 Baum, E. *et al.* Submicroscopic and asymptomatic *Plasmodium falciparum* and *Plasmodium vivax* infections are common in western Thailand - molecular and serological evidence. *Malar. J.* **14**, 95, doi:10.1186/s12936-015-0611-9 (2015).

3 Shahzadi, S. *et al.* Molecular detection of malaria in South Punjab with higher proportion of mixed infections. *Iran. J. Parasitol.* **9**, 37-43 (2013).

4 Golassa, L. *et al.* Microscopic and molecular evidence of the presence of asymptomatic *Plasmodium falciparum* and *Plasmodium vivax* infections in an area with low, seasonal and unstable malaria transmission in Ethiopia. *BMC Infect. Dis.* **15**, 310, doi:10.1186/s12879-015-1070-1 (2015).

5 Tripura, R. *et al.* Persistent *Plasmodium falciparum* and *Plasmodium vivax* infections in a western Cambodian population: implications for prevention, treatment and elimination strategies. *Malar. J.* **15**, 181, doi:10.1186/s12936-016-1224-7 (2016).

6 Steenkeste, N. *et al.* Sub-microscopic malaria cases and mixed malaria infection in a remote area of high malaria endemicity in Rattanakiri province, Cambodia: implication for malaria elimination. *Malar. J.* **9**, 108, doi:10.1186/1475-2875-9-108 (2010).

7 Imwong, M. *et al.* The epidemiology of subclinical malaria infections in South-East Asia: findings from cross-sectional surveys in Thailand-Myanmar border areas, Cambodia, and Vietnam. *Malar. J.* **14**, 381, doi:10.1186/s12936-015-0906-x (2015).

8 Nguitragool, W. *et al.* Very high carriage of gametocytes in asymptomatic low-density *Plasmodium falciparum* and *P. vivax* infections in western Thailand. *Parasit Vectors* **10**, 512, doi:10.1186/s13071-017-2407-y (2017).

9 Lover, A. A. *et al.* Prevalence and risk factors for asymptomatic malaria and genotyping of glucose 6-phosphate (G6PD) deficiencies in a vivax-predominant setting, Lao PDR: implications for sub-national elimination goals. *Malar J* **17**, 218, doi:10.1186/s12936-018-2367-5 (2018).

10 Chaturvedi, N. *et al.* Prevalence of afebrile parasitaemia due to *Plasmodium falciparum* & *P. vivax* in district Balaghat (Madhya Pradesh): Implication for malaria control. *Indian J Med Res* **146**, 260-266, doi:10.4103/ijmr.IJMR_1538_16 (2017).

11 Starzengruber, P. *et al.* High prevalence of asymptomatic malaria in south-eastern Bangladesh. *Malar. J.* **13**, 16, doi:10.1186/1475-2875-13-16 (2014).
